# “*It is very difficult in this business if you want to have a good conscience*”: pharmaceutical governance and on-the-ground ethical labour in Ghana

**DOI:** 10.1080/11287462.2022.2103899

**Published:** 2022-07-26

**Authors:** Kate Hampshire, Simon Mariwah, Daniel Amoako-Sakyi, Heather Hamill

**Affiliations:** a Durham University, Durham, UK; b University of Cape Coast, Cape Coast, Ghana; c University of Oxford, Oxford, UK

**Keywords:** Access to medicines, essential medicines, pharmaceutical governance, Africa, ethical labour, substandard and falsified (SF) medical products

## Abstract

The governance of pharmaceutical medicines entails complex ethical decisions that should, in theory, be the responsibility of democratically accountable government agencies. However, in many Low- and Middle-Income Countries (LMICs), regulatory and health systems constraints mean that many people still lack access to safe, appropriate and affordable medication, posing significant ethical challenges for those working on the “front line”. Drawing on 18 months of fieldwork in Ghana, we present three detailed case studies of individuals in this position: an urban retail pharmacist, a rural over-the-counter medicine retailer, and a local inspector. Through these case studies, we consider the significant burden of “ethical labour” borne by those operating “on the ground”, who navigate complex moral, legal and business imperatives in real time and with very real consequences for those they serve. The paper ends with a reflection on the tensions between abstract, generalised ethical frameworks based on high-level principles, and a pragmatic, contingent ethics-in-practice that foregrounds immediate individual needs – a tension rooted in the gap between the theory and the reality of pharmaceutical governance that shifts the burden of ethical labour downwards and perpetuates long-term public health risks.

## 1. Introduction

### 1.1 Access to medicines and the challenges of pharmaceutical governance

Ensuring access to safe, affordable, appropriate medicines is central to achieving Universal Health Coverage, a key tenet of the health-related Sustainable Development Goals (United Nations, n.d.). As Kohler et al (2014:3), among others, have noted, “The efficient and safe selection, procurement, storage and delivery of pharmaceutical commodities are essential functions in ensuring sustained global health outcomes, especially in resource-poor settings.” In most countries, these responsibilities are distributed between various state and non-state actors. For example, Ministries of Health and health service providers (public, private and voluntary sector) are tasked with procuring, purchasing and delivering pharmaceutical medicines to health facilities and patients; while National Medicine Regulatory Authorities (NMRAs), including Food and Drugs Authorities, Standards Authorities, etc., oversee licencing and quality assurance; and professional organisations are responsible for ensuring that those prescribing and dispensing medicines are properly trained and adhere to professional codes of practice. Collectively, we refer to these tasks and responsibilities as “pharmaceutical governance”, drawing on the concept of governance as “managing the resources and affairs of society to promote the well-being of its members” (Anello, 2008).^1^

Governance of the pharmaceutical sector, especially in Low- and Middle-Income Countries (LMICs), presents a unique set of challenges (Kohler et al, 2014). A combination of limited regulatory capacity , the size and complexity of global supply chains (Mackintosh et al., 2011; 2018; Tremblay, 2013), and the strong financial incentives for manufacturers and traders to cut corners, make full control of the supply side difficult to achieve. On the demand side, a growing double burden of disease (with the rapid rise of chronic non-communicable disease alongside infectious disease) and widespread poverty continue to fuel a high unmet demand for affordable, effective drugs. The sheer volume of medicines moving around the world is immense, with annual global spending on pharmaceuticals currently exceeding USD 1.3 trillion and projected to approach USD 1.6 trillion by 2025 (Statistica, 2020 data). However, according to the WHO, only 30% of NMRAs globally currently have the capacity to effectively evaluate the quality, safety and efficacy of medical products on the market (Ndomondo-Sigonda et al., 2017; WHO, pers. comm.). In Africa, only two NMRAs (Tanzania in 2018 and Ghana in 2020) have attained WHO Maturity Level 3 (“stable, well-functioning and integrated regulatory systems”), and none have reached Level 4 (“regulatory systems operating at advanced level of performance and continuous improvement”) (WHO, 2021).

As a result of these challenges, large numbers of people in LMICs still lack access to safe, affordable, appropriate medicines. Drawing on WHO data, Bigdeli et al. (2018) report that availability of generic essential medicines^2^ in the public sector in LMICs ranges between 38% and 46%. Patients are thus obliged to turn to the private sector, where availability is somewhat better (c. 70%) but prices tend to be higher and unaffordable for many. Safety is another significant concern. The WHO recently estimated that over 10% of medical products in LMICs are substandard or falsified (SF)^3^, failing to meet internationally-recognised quality standards (WHO, 2017a; 2017b; see also Ozawa et al., 2018). Such products, which may contain little/no active ingredient, the wrong active ingredient or, in some cases, toxic substances, are reckoned to cause over 150,000 childhood deaths each year from malaria alone and a similar number from pneumonia (Nayyar et al., 2019; Renschler et al., 2015; WHO, 2017a), with the poorest disproportionately affected (Evans et al., 2019). Finally, it is estimated that, worldwide, more than 50% of all medicines are prescribed, dispensed, or sold inappropriately: the wrong drug for the patient’s clinical needs, in the wrong dosage, and/or for the wrong duration (WHO, nd(b)). In addition to the health risks and financial costs for individual patients, the combination of over-prescribing antimicrobial medicines (antibiotics, antimalarials, etc.) and under-dosing (where patients cannot afford a full course of medication) is a particularly pernicious one, contributing significantly to the unfolding global public health crisis of antimicrobial resistance (WHO, 2015). Similar concerns have been raised about the impact of SF medicines containing sub-therapeutic amounts of antimicrobial ingredients (Newton et al., 2010; 2016).

### 1.2 The ethics of pharmaceutical governance in resource-limited settings

Even in High-Income Countries (HICs), pharmaceutical governance entails complex ethical questions and decisions around the efficacy, safety and cost-effectiveness of treatments. For example, in England and Wales, NICE (the National Institute for Health and Care Excellence) makes decisions about how to allocate finite resources between competing demands and priorities, with controversies often arising about the value of expensive medications; for example, end-of-life cancer drugs, which can offer patients a few extra months of life at costs of tens of thousands of pounds (The Guardian, 2008, 2017).

In LMICs, such decisions and associated trade-offs may come into play at far lower thresholds, as governments struggle to provide even basic essential medicines for their populations. Pisani (2019) has recently suggested that pressures to meet Universal Health Coverage targets could result in a lowering of drug quality, as governments seek to source cheap medicines, driving down effective quality assurance. In a recent discussion piece, Ravinetto et al. (2018, p. 83) asked “whether it could ever be ethically justified to compromise on the quality assurance of medicines depending on what individuals, communities, or societies can afford”. This is an uncomfortable question because it poses a direct challenge to the four widely-accepted and supposedly “universal” principles of medical ethics (Beauchamp & Childress, 2013): respect for autonomy, beneficence (doing good), non-maleficence (avoiding harm), and justice. As such, it raises the deeply problematic possibility that we should accept “different [ethical] standards for the advantaged and the disadvantaged”, as Farmer and Gastineau (2004, p. 246) put it.

Ravinetto et al concluded that

“there is a moral obligation that, under any circumstances, due attempts should be made to apply the best possible quality assurance methods and standards, in order not to expose patients to possible harm from non-quality-assured medicines or unsecured supply channels” (Ibid:90),

with exceptions admissible only in “*exceptional* and *temporary* circumstances, if based on a meaningful quality risk assessment, guided by a rigorous ethical framework built on the principles of independence, technical competence, transparency, and accountability” (Ibid:81, italics in original). They propose that “an international group of experts in quality assurance/regulatory affairs and health ethicists” be established to oversee and formulate appropriate ethical responses. While it is difficult to argue against this conclusion, the ideal of an “international group of experts” making well-informed, independent assessments is a far cry from the current reality of over-stretched, under-resourced regulatory agencies typical of many LMIC contexts (Hamill et al., 2021). In practice, as we show below, many decisions are taken by people working “on the ground”, far away from government offices where standards and protocols are drawn up. Such decisions may have to be made quickly, with limited information, and in relation to “real people” whose health (and even lives) might be at stake.

A small but growing body of recent work seeks to understand how, in resourced-constrained healthcare contexts, individuals in positions of responsibility attempt to reconcile (or otherwise manage) a gap between what they *ought* to do and what they *can* realistically do, given the situations they find themselves in. For example, Wendland (2010) described the agonising incongruity faced by medical students in Malawi in trying to reconcile universal “clinical best practice” with the reality of working in a hospital lacking the most basic equipment. Livingston (2012), recounting a similar situation in a Botswana oncology ward, highlighted the “improvisation” required of health professionals managing end-of-life cancer care in the absence of any effective pain relief. Similarly, in Cambodia, Gryseels et al. (2019) have drawn attention to the tensions faced by healthcare professionals between adhering to “an imagined [international] medical ‘standard'” and the situation on the ground (inadequate healthcare funding and infrastructure, poor regulation, patient demand for cheap and “quick acting” drugs) that “compel[s] improvisation and divergence” from these standards.

These are ethical challenges that cannot easily be delegated to a distant review board or “international group of experts”; they have to be managed *on the ground* and *in the moment*. Writing about a medical research institution in Western Kenya, Kingori (2013) described the “ethical labour” undertaken by local data collectors working on the “front line” in a context of high disease burdens, poor healthcare provision and grinding poverty. These data collectors were caught between two very different sets of moral imperatives: those of the organisations they represented, which required compliance with ethical standards such as non-intervention, and those of the communities in which they lived and worked, which required them to act if someone needed medical attention. Drawing on Ricoeur’s (1992) earlier work, Kingori showed that “everyday ethics” are deeply embedded in social relations that depend not just on “the what” and “the why” but also on “the who”. Similar tensions have been described by Häggström et al. (2008), writing about the work of nurses in Tanzania, and by Fisher (2006) in relation to clinical trials in the US. In each case, those working on the ground had somehow to balance their own (and their communities’) moral understandings with institutional policies and frameworks governing clinical decision-making or “human subjects research”: a significant burden of “ethical labour” that those institutional frameworks did little to support or mitigate.

### 1.3 Aims and outline of this paper

As we have suggested above, the governance of pharmaceutical medicines entails complex ethical questions and decisions that should, in theory, be the responsibility of democratically accountable government agencies (sometimes in partnership with non-state actors). However, it is clear that, particularly in LMICs settings, there are significant challenges in high-level pharmaceutical governance that expose patients to multiple risks. In this paper, we describe and reflect on the resulting “ethical labour” that is transferred to those involved in the day-to-day business of buying, selling and regulating medicines lower down the supply chain in Ghana: a West African country with relatively well-functioning regulatory authorities.

The paper proceeds as follows. After a brief description of the study context and methods, we outline the practices and challenges inherent in pharmaceutical governance in Ghana, focussing on three main areas: distribution and supply, regulation of drug quality and safety, and regulation of professional practice. As we will show, despite relatively robust institutional frameworks, those inside the system recognised significant challenges. We then present three detailed case studies of individuals operating lower down the supply chains: an urban retail pharmacist, a rural over-the-counter medicine retailer, and a local inspector, who must navigate complex moral, legal and business imperatives in real time and with very real consequences for those they serve. The paper ends with a reflection on the tensions between abstract, generalised ethical frameworks based on high-level principles, and a pragmatic, contingent ethics-in-practice that foregrounds immediate individual needs – a tension rooted in the gap between the theory and the reality of pharmaceutical governance that shifts the burden of ethical labour downwards and perpetuates long-term public health risks.

## 2. Study setting and methods

This paper draws on 18 months of ethnographic fieldwork undertaken in Ghana (2016–17) by the authors and research assistants as part of the “Trust in Medicines” (TrustMed) project, funded by the Wellcome Trust. Ethical clearance was granted by IRBs at the Universities of Cape Coast, Durham and Oxford.

Ghana represents an interesting case study for this work. It is the second largest manufacturer of pharmaceutical medicines in West Africa (after Nigeria), with over 30 licensed manufacturers, supplying around 30% of medicines in the country. The remaining 70% are imported, mostly from Asia (especially India and China) and Europe, with smaller quantities coming from elsewhere in Africa. Ghana’s regulatory authorities are regarded as among the most effective in Africa (PMQ, 2016); as noted above, its Food and Drugs Authority (FDA) recently became the second in Africa to attain WHO Maturity Level 3, indicating a “well-functioning system”. Ghana was also a regional forerunner in extending health coverage via its National Health Insurance Scheme (NHIS), introduced in 2003, the first of its kind in Sub-Saharan Africa (Agyepong & Adjei, 2008). Nonetheless, as we discuss further below, there remain significant challenges in ensuring that Ghana’s population have access to safe, appropriate and affordable medicines. Despite significant investment, NHIS coverage remains limited at c. 35% (Nsiah-Boateng & Aikins, 2018), and public-sector stock-outs of essential medicines persist (Masters et al., 2014; Poku et al, 2017). The country has also experienced a high reported prevalence of SF medicines: 35–37% for antimalarials at the time of our fieldwork (Kaur et al., 2016; Tivura et al., 2016).

Fieldwork for this study was conducted in Greater Accra (in/around the capital city) and in Central Region (in/around of Cape Coast, some 100–150 km west of Accra). Over 200 interviews and accompanying ethnographic observations were conducted with individuals and organisations operating at different points in pharmaceutical supply, retail and regulation in the country (see also authors, 2019; 2021). In this paper, we draw specifically on our interactions with licenced retail pharmacists (N = 25) and licenced over-the-counter (OTC) medicine retailers (N = 40) based in Greater Accra and Central Region (rural and urban settlements), plus 20 key informant interviews with national- and regional-level representatives of organisations involved with pharmaceutical governance in Ghana (including Food and Drugs Authority, Pharmacy Council, Ghana Health Service and others).

Interviews were conducted in either English or Twi, according to participants’ preference; they were wide-ranging, loosely structured around themes of medicine quality, distribution and regulation, allowing interviewees considerable scope to direct the conversation. As in any study based on self-reporting, we recognise the risk of “social desirability bias” (Bergen & Labonté, 2020) whereby interviewees may seek to present themselves in a favourable light. In this case, we were asking not just about *undesirable* behaviours but potentially incriminating *illegal* activities. However, the vast majority of our study participants appeared to be remarkably open and frank in our discussion. Indeed, many seemed to find some relief in sharing their experiences in a confidential forum.

Detailed hand-written notes were taken during the interviews^4^ and were typed up shortly afterwards (those in Twi were translated into English). Data analysis followed the principles of Grounded Theory, whereby theoretical insight emerges from the data through an inductive process of close-reading, coding and testing of nascent hypotheses through subsequent fieldwork (Corbin & Strauss, 2015). Through this process, ethical dilemmas emerged as a prominent theme, especially for those working “on the ground”, interacting with patients.

Below, we begin by outlining the practices and challenges inherent in pharmaceutical governance in Ghana, as depicted by representatives of key organisations. We then present three extended case studies selected purposively to illustrate the range of daily ethical quandaries encountered by our participants: a retail pharmacist in Greater Accra, a small-scale OTC medicine retailer in rural Central Region, and a Pharmacy Council inspector, also based in Central Region. Case studies are a well-recognised way of presenting qualitative research findings (Yin, 2014). We use them here because they allow the reader to enter more deeply into participants’ worlds and to understand how ethical decision-making is contextualised in practice. Because of the sensitivity of some of the information presented, we have taken additional care to protect interviewees’ anonymity and confidentiality by changing names and other potentially identifying features; representatives of high-level agencies, whose identification might be particularly problematic, are referred to simply as “study participants”.

## 3. Pharmaceutical governance in Ghana: practices and challenges

As in other countries, the task of pharmaceutical governance in Ghana is distributed across multiple state and non-state agencies. Procurement, purchase and distribution of medicines to public-sector health facilities is managed by the Ghana Health Service, through its Central Medical Store, operating under the Ministry of Health. Private- and voluntary-sector providers follow their own procurement and distribution practices; notable among these is the Christian Health Association of Ghana (CHAG), the second largest healthcare provider in Ghana, with nearly 350 facilities serving the most remote rural areas.^5^ Regulatory responsibility for assuring the safety, quality and efficacy of medicines in Ghana falls to the Food and Drugs Authority (FDA), who are mandated by law to licence drugs, inspect production facilities to ensure Good Manufacturing Practice (GMP), monitor the quality of products entering the country, and conduct regular Post-Market Surveillance (PMS). Complementing their work, the Pharmacy Council oversees and regulates professional conduct in pharmacies and over-the-counter (OTC) outlets. Other agencies, including the Ghana Standards Authority, Ghana Revenue Authority and the Ghana Police Service are also involved in identifying and intercepting suspect products at ports of entry and may assist in post-market surveillance.

While representatives of these organisations were generally keen to showcase good practice, almost all acknowledged significant challenges, leading to some concerns about the availability, safety and/or appropriateness of medicines reaching patients. We elaborate on each of these below.

### 3.1 Distribution and supply

The Ghana Health Service is responsible for ensuring the supply of medicines on the National Drugs List to all public-sector facilities across the country. In theory, Regional Medical Stores (RMS) are supplied by the National Medical Store (NMS) and, in turn, they supply facilities in their region. However, according to one study participant, chronic shortage in official supplies often forces Regional Stores to source medicines directly from the “*open market*” through an expedited process with less stringent controls: “*We go shopping*”, he put it. Similar concerns were expressed by a representative of a voluntary-sector organisation also supplied by Regional Medical Stores:

“*We cannot avoid sourcing from the open market if RMS has stock-out. […] Regulation of the open market is a big challenge. […] It’s very difficult to check the registration status of suppliers, especially if you’re in a hurry because you’re in need*.”

Even then, demand from facilities tends to exceed supply, with stock-outs of essential medicines a reportedly common occurrence. As one interviewee admitted, “*Nothing is adequate. We are constrained. We have to admit it*.” Some also blamed alleged “leakages” of government-purchased medicines which found their way into private retail outlets; a practice reported elsewhere in West Africa, driven by shortages, low wages and poor governance (Onwujekwe et al., 2019).

In cases of public-sector stock-outs, patients are directed to fill prescriptions at private pharmacies. Private pharmacies were often said to be more expensive, stocking high-end generics or proprietary medicines that were unaffordable for many. Long delays in reimbursements were blamed for more and more private pharmacies opting out of the National Health Insurance Scheme, obliging customers to meet the costs out of pocket (see also Akweongo et al., 2021). Moreover, as several interviewees acknowledged, pharmacies were few and far between in rural areas, especially in the northern regions.

### 3.2 Regulation of drug quality and safety

Assuring the safety and quality of medicines was another major area of concern. Many interviewees drew attention to the size and complexity of pharmaceutical supply networks in Ghana, which made regulation challenging. As one study participant put it, “*There are too many wholesalers; too many big companies which means the supply chain is not well organised. It is too fragmented and difficult to control.*”

With 70% of medicines coming from outside the country, several interviewees believed that Ghana’s “*porous borders*”, especially the eastern border with Togo, increased susceptibility to penetration of poor-quality products. One representative told us,

*“A lot of medicines enter the country illegally through Togo from Nigeria. Nigeria is the hub of fake drugs – I’m sorry to say it, but it’s true. There are big cartels operating there. We imported huge quantities of amoxicillin but it contained no API [active pharmaceutical ingredient]!”*.

Another reported the recent interception of a truck from Togo carrying an “*illegal consignment of antimalarials and painkillers”*, noting also the practice of people crossing the border on foot, head-loading cartons of unregistered medicines. (2016 #2). Some also cast doubt on the moral integrity of importers who were, they believed, “*only interested in making money*”. A couple of interviewees even mentioned the possibility of collusion between overseas manufacturers and Ghanaian importers to reduce costs by reducing the amount of active ingredient (API) in certain products.

Given this context, it was widely acknowledged that, however well-intentioned, regulatory agencies lacked the capacity (human, financial and technical resources) to do a comprehensive job. One study participant was frank in his view that, despite rigorous protocols, the FDA’s drug enforcement team was simply too small for the work required. Others pointed to perceived gaps in other areas, including post-market surveillance (PMS): “*The FDA do a good job on testing the quality of products when first imported but their ability to do PMS [Post-Market Surveillance] is limited*,”, or the capacity of the FDA to ensure Good Manufacturing Practice in overseas facilities: “*The biggest problem by far is imported generics. They get a 3-year licence from FDA, with very little checking after the preliminary samples. The FDA are completely overwhelmed by PMS […] They really don’t have the capacity*.” Similar concerns were raised about enforcement agencies, including the Ghana Revenue Authority and the Ghana Police, reinforcing the perception that those breaching regulations were rarely caught or faced penalties.

### 3.3 Regulation of professional practice

A third area of concern was around professional practice of those dispensing and selling medicines; this falls under the jurisdiction of the Pharmacy Council, a statutory body charged with ensuring that “*competent pharmaceutical care providers who practice with agreed standards are accessible to the whole population*” (Pharmacy Council of Ghana, n.d.). Responsibilities of the Council include overseeing the training and continuous professional development of pharmacists and over-the-counter (OTC) medicine retailers, registering practitioners, licencing retail outlets and carrying out regular inspections to ensure that medicines are stored and dispensed appropriately, “rationally”, and in line with the terms of their licence.

Again, however, concerns were raised about regulatory effectiveness, particularly around inspections. For example, one study participant told us:

“*The Pharmacy Council has a lot on their head: inspecting new pharmacies, current ones and monitoring. They don’t have enough staff, so they may only pass through each pharmacy once a year. And people alert each other if the Pharmacy Council are on their way. […] Over-the-counter medicine shops are only supposed to sell class C drugs – not antibiotics and no POM [Prescription Only Medicines]. But if people don’t get what they demand from pharmacies, they go and get it from OTC shops. […] There is no strong punishment if there is found to be no pharmacist present in a pharmacy. The fine is just GHS 3500. But it costs GHS 3000 per month to employ a pharmacist! So the regulator is not punishing people enough. […] One pharmacy I know of had no pharmacist for 10 months, so they saved around GHS 30,000, and only had to pay a GHS 3500 fine. […] Last time there was monitoring [names area], only 32% of pharmacies had a pharmacist present. The regulator says they are under-resourced and that’s why they can’t do enough inspections.”*

Another capacity issue raised by some was the long backlog of processing licence and renewal applications; a situation which, in conjunction with escalating fees, was seen as a barrier to compliance.

In summary, although Ghana has some of the most effective regulatory authorities in Africa, key actors from all of these agencies (and other high-level stakeholders) freely admitted that financial, technical and human resource constraints limited what they could realistically achieve. In this context, individuals operating further down the supply chains must make some difficult decisions, as we show through the following case studies.

## 4. Managing medicines on the ground: three case studies

### 4.1 Joseph: urban retail pharmacist^6^

Joseph proudly showed us into his pharmacy: a large, newly-constructed building in a suburb of Accra. Inside, it was strikingly empty. A modest array of medicines were arranged neatly on shelves. Behind was a small “consultation room” with a blood pressure monitor sitting on a small table. Joseph explained that he was just starting up; he had ambitious plans to expand if things went well.

Joseph grew up in Ghana, but had spent most of his adult life in Europe, where he trained and then worked as a hospital pharmacist. However, his “life-long dream”, he said, was to return “home” to set up his own pharmacy in Ghana. For the last three years he had travelled between Europe and Ghana, overseeing construction of the premises on a plot of land he had bought for the purpose. Upon retirement (three months prior to our interview), he moved to Accra to “get the business going”, leaving his wife and children in Europe. However, “getting going” was proving more difficult than anticipated. Trade was slow; in the two hours we spent there, not a single customer came by. Joseph talked despondently about the competition from a new pharmacy just down the road.

Conversation turned to the issue of medicine quality and the difficulties of sourcing trustworthy products. Joseph explained:

“*It’s very difficult to identify genuine items. The issue of sourcing medicines here is very complex because there are no specific supply chains. There are so many wholesalers.*^7^ *[…] The wholesalers come round, and the packaging looks perfect but packaging alone is not enough to distinguish a genuine medicine. […] The lack of regulation makes it more or less impossible to be sure about medicine quality*.”

Initially, Joseph opted for the lower-cost products, reasoning that, in the absence of other information, at least his medicines would be more affordable:

“*Sometimes I would just go for price – I would take the cheapest one, because there is actually no way to tell which medicines are the quality ones. Even if it’s just 20 pesawas*^8^ *cheaper than the other one, it means I can pass the savings onto the customer and they will start coming to me*.”

However, a particular incident changed his views. One day, a customer complained that his flucloxacillin capsules were not working. When Joseph opened a capsule, he was shocked:

“*It should be a powder but it had solidified into a solid lump, like a stone. Anyone taking that medicine could not get cured. I reported it to the wholesaler and, when we checked, we found the whole consignment was the same*.”

The wholesaler then became angry and threatening.

Shaken by this episode, Joseph decided to switch to the UK brands he knew and trusted. However, these were unaffordable for many customers:

“*Now I only buy the UK brands of flucloxacillin, even though it is much more expensive: GHS 25 rather than GHS 5 for a 7-day course. The problem is people don’t want to spend that much money, so they just go to a different place [waves his arm towards the nearby pharmacy]*.”

Moreover, he is still reliant on buying from local wholesalers whom he trusts less and less:

“*I’ve heard that some of these people can go to India and negotiate with manufacturers to supply them with different qualities of drugs depending on what money they have. So, if they go with less money, they can negotiate to buy low-quality medicines with less active ingredient. The government doesn’t have a good means of checking.*”

The other option is importing directly, as Joseph explained: “*The only way to be one hundred per cent sure is to import directly from the UK.*” To do this legally, however, is expensive and requires “*a lot of bureaucracy”*. Instead, he opts to bring what he can in his suitcase. Price remains an issue:

“*The one I bring from UK may cost GHS 35 [c. GBP 7], but the customer comes with only 1 GHS and cannot pay any more. So, I give them the Ghanaian medicine, which is very cheap, but I feel very uncomfortable because I don’t really know how good it is*.”

Joseph’s dilemmas were shared by other pharmacists we interviewed, who struggled to reconcile customers’ need for efficacious medicine with their inability to pay for (supposedly) higher-quality products, combined with the uncertainties of an under-regulated supply chain. Losing customers to competitors represented both a financial risk and a moral risk. Joseph reasoned that, if the other pharmacy was selling cut-price medicines, they were unlikely to be of good quality. It was only by carefully balancing pricing, legality and following his “hunches” that Joseph tried to protect both his business and his customers’ health.

Towards the end of our interview, Joseph brought out two bottles of a UK-branded “tonic”: he had purchased one from a local wholesaler; the other he had brought from the UK. The packaging was very similar but not identical. There were small differences in the size and position of the logo, which made him wonder: had the manufacturers simply changed the packaging or had he been sold a “fake”? Should he take it up with the wholesaler? Based on his previous experiences, they were unlikely to respond sympathetically. Should he alert the authorities? He had no “insider connections” and very little confidence that they would take it seriously. He also feared the reputational damage if word got round, undermining his (already fragile) client base and perhaps bringing repercussions from wholesalers who may not be willing to deal with him again. Should he dispose of the locally-purchased batch and sell only those he had brought directly from the UK? What about customers who couldn’t afford the UK-purchased ones? And what to do when they ran out? There were limits to how often he could travel to the UK and how much he could fit in his suitcase, apart from the dangers of getting stopped at customs. Such quandaries weighed heavily on Joseph. He sighed, “*You lose customers by trying to help them. It is very difficult in this business if you want to have a good conscience*.”

### 4.2 Isaac – rural over-the-counter medicine retailer

Isaac’s small over-the-counter (OTC) medicine shop in a remote village in Central Region was a far-cry from Joseph’s up-market urban venture. Isaac had started out some 30 years previously as an itinerant medicine vendor, buying from a large retail pharmacy and re-selling (without a licence) in rural settlements. After several years, he was offered a position dispensing medicines at the pharmacy, where he learned a lot “on the job”. Eventually, he saved enough money to establish his own small OTC shop in a village where his brother, the Assemblyman (an elected local government representative), helped him with the necessary paperwork and up-front registration costs. Business was slow, however, and he relied on farming to supplement his income. Five years before our interview, he moved to his current location, where the farming was more profitable. It was relatively easy, he said, to get his business established in the new setting because people remembered and trusted him from his time as an itinerant seller.

When Isaac first set up the shop in the village, there was no other licensed medicine outlet. Since then, a small government clinic (a CHPS [community-based health planning and services] compound) was established. However, according to Isaac, the clinic rarely has adequate medicine supplies; instead, patients are given a prescription to fill elsewhere. The nearest pharmacy is an hour’s drive down a heavily pot-holed dirt road: an expensive journey on infrequent public transport and often impassable in the rainy season. Isaac therefore has a steady stream of patients turning up with prescriptions for antibiotics and other medicines that he is not permitted to stock under terms of his OTC licence.

At first, Isaac explained, he would take his customers’ prescriptions to fill on their behalf in town. However, this strategy quickly became untenable: either patients would have to wait several days until he was going to town, or he would have to make a special trip, which was expensive (he could not pass the extra cost onto the customer) and meant leaving his shop unattended. Isaac saw that some customers began resorting instead to itinerant peddlers who undercut his prices and whom he saw as unreliable: “*they can just run away*” if things go wrong. Eventually, he used his “connections” to purchase and stock a small range of prescription-only antibiotics for common skin and respiratory infections, risking legal penalties in order (as he saw it) to protect both his business and his customers’ health. During our interview, a young woman arrived at the shop with a large, suppurating wound on her arm. She handed a prescription to Isaac, for flucloxacillin, paracetamol and vitamin C tablets. He dispensed all three products, explaining carefully – and accurately – what each was for and how it should be taken.

In the year or so prior to our interview, two new OTCs have opened in the village, further compelling Isaac to meet customer demand for prescription medicines: if he doesn’t supply them, his competitors will.

### 4.3 Mensah – Pharmacy Council inspector

In a small district office, Mensah told us about his work, supporting the Pharmacy Council’s mandate to “*promote the rational use of properly registered medicines*” and to “*curb the menace”* of unlicensed vendors. In his role as an inspector, Mensah regularly visited pharmacies and OTC retailers in his catchment area, checking licences and ensuring that medicines for sale complied with regulations (in-date, approved for sale in Ghana, within terms of the outlet licence, etc.). Occasionally, he would also be involved in “swoop operations” targeting unlicensed vendors.

Mensah described the penalties for regulatory infringements, ranging from fines to temporary or even permanent closure for more significant breaches. However, this left Mensah with a quandary. As a local resident himself, he recognised the constraints that many small-scale retailers were operating under, which could make it difficult to comply fully with regulations. For example, he knew that the process of obtaining and maintaining a licence could be lengthy and expensive, particularly for those without the “right connections”. And he understood why OTC retailers might sometimes be tempted to stock prescription-only medicines or sell individual doses to customers who are unable to afford a full course.

It was thus important to “*balance strategically*” (as Mensah put it) the healthcare needs of a community with regulatory requirements. In some cases, the decision was straightforward. For example, Mensah had had few qualms about ordering the temporary closure of an OTC outlet in an urban area staffed only by a 14-year-old boy:

“*In that case, it was not safe to have a boy in charge, and because it is a town with other retailers nearby, it would not be detrimental to the community to shut this one down temporarily.*”

When other options were not available, however, it could be a different matter:

“*If the medicine seller is operating in a rural community, where there is nowhere else for people to buy medicines, it is better not to arrest them but to work with them to get them properly certified. Otherwise, you may have a community of 1000 people with no way of buying any medicines.*”

Timing was everything in such cases. Rather than order the closure of an outlet pending training or proper certification, Mensah was often inclined to support shop-keepers to remain open for business while they (hopefully) got their papers in order, to avoid people going without medicines at all or turning to riskier sources. “*If a village ends up not having an approved medicine outlet, then more people will end up getting drugs from peddlers,*” he explained, acknowledging this sometimes meant turning a blind eye to some infringements indefinitely.

## 5. Discussion

On the face of it, the challenges and dilemmas experienced by Joseph and Isaac are typical of those faced by any other small-scale business owners operating on tight margins in a difficult, uncertain and under-regulated market. However, unlike sellers of leather belts, stereo systems and other goods, medicine retailers in our study were acutely aware that the decisions they made could have very serious, even existential, consequences for their customers, many of whom they knew personally as friends and neighbours. Similar considerations applied to Mensah and other local inspectors, who often had long-standing relationships with the communities they worked in and were acutely aware that, especially in rural areas, making the wrong decision could deprive a whole village of access to medicines.

The experiences of Joseph, Isaac, Mensah and others were far removed from the ideal set out by Ravinetto et al. (2018, p. 81), in which complex decisions about pharmaceutical supply and quality control should be taken on the basis of a “rigorous ethical framework built on the principles of independence, technical competence, transparency and accountability.” Instead, like the Kenyan data collectors (Kingori, 2013) and Tanzanian nurses (Häggström et al., 2008), our interlocutors were responding on a daily basis to the pressing needs of real individuals, whose experiences of poverty and vulnerability they shared. As such, they are motivated first and foremost, not by abstract principles, but by the *practical and immediate outcomes* of their actions in *specific situations*. Drawing on a deep (often embodied) contextual understanding, they were able to act on “hunches” and intuitions on a case-by-case basis (for example, an assessment of an individual’s personal circumstances and options available to them, or a community’s need for a trusted – if not fully compliant – OTC retailer).

Of course, these decisions were not *just* ethically motivated. As business owners and employees, they had to try to reconcile and somehow balance the urgent health needs of those around them with their professional and legal obligations, their personal business interests, and their own “*consciences*” (as Joseph put it). Sometimes, these moral, legal and business imperatives might align; for example, when Mensah closed down the urban pharmacy staffed by a young boy, he was acting both legally and morally (removing what he believed to be a less trustworthy retailer in the knowledge that other options were available for local residents). Oftentimes, however, they come into direct conflict with one another, with individuals breaking the law in various ways (illegally importing medicines from Europe; selling prescription-only medicines from an OTC outlet) in order to keep their businesses afloat and to serve (as they saw it) the needs of their communities. There are also many “grey areas”, where the blurred line between commercial interests and ethical concerns could potentially generate what Feldman and Halali (2019) have called “subtle conflicts of interest”. For example, Isaac’s decision to stock prescription-only medicines, and Joseph’s attempts to bring medicines from the UK, were represented as moral acts (in making higher-quality medicines available to people who might otherwise use less trustworthy sources) but also made sound business sense.

The key point here is that these ethical quandaries and “grey areas” come as a direct result of challenges in high-level pharmaceutical governance. Had there been more effective regulation of wholesalers and their products, and easier ways to report suspicions, Joseph might not have felt obliged to turn to illegal “suitcase importing”. Had there been a licenced pharmacy within reach of people in Isaac’s village, or if supplies of essential medicines in the local clinic were adequate, Isaac might not have felt obliged to illegally sell antibiotics and Mensah might have had fewer qualms about imposing penalties on retailers like Isaac. This is not to say that our interlocutors *would not* have acted illegally in these circumstances; in every country, some people break the law for personal gain. However, the grey areas would have been fewer and it would have been harder to justify acting illegally on ethical grounds.

What are the consequences of this situation? In some ways, the informal system represents a pragmatic response to a challenging set of circumstances. Assuming that *most* medicine retailers and local inspectors are reasonably well-intentioned and well-informed, and that the *majority* of medicines dispensed are of decent quality, then current practices probably mean that more people are getting the medicines they need than might otherwise be the case, and are avoiding the riskiest sources. On average, those medicines will probably do more good than harm, leading to better health outcomes and potentially saving lives.

However, there are significant risks, both for those working on the “front line” and for wider public health. First, there is the threat of legal penalties. Although rarely imposed, Joseph and Isaac could face heavy fines and the closure of their businesses for acting as they do. Likewise, Mensah could be sanctioned if his superiors found out that he was “turning a blind eye” to certain infringements (although they might well be doing the same). Perhaps more significant, though, is the burden of ethical labour, borne disproportionately by those “on the ground” (cf Kingori, 2013). This is in some ways reminiscent of “task shifting” in healthcare: the redistribution of tasks from more-qualified to less-qualified health workers, advocated by the World Health Organisation as a “*pragmatic response to health workforce shortages*” (WHO, 2008, p. 3; Zachariah et al., 2009), but criticised by others as unfairly over-burdening poorly-paid community health workers (Smith et al., 2014). The same could be said for the downward shifting of the ethical labour of pharmaceutical governance from highly-paid policy-makers and regulators to those working on the ground, operating on minimal margins, and bearing the moral, professional and legal risks individually.

There are also significant longer-term public health risks. Although individuals like those profiled above may be acting (as they see it) in the interests of those in their communities by maintaining access to less risky medicine sources, collectively, their actions may serve to undermine the integrity of pharmaceutical supply chains and confound regulatory efforts. The aggregated actions of decisions that may be reasonable (and apparently “ethical”) at the individual level can have seriously detrimental systemic effects, as complex systems modellers have shown (see Ackland et al., 2019, for an example relating to medicine quality). In this case, those effects may serve to perpetuate, and even exacerbate, pre-existing health inequalities, since it is disproportionately poorer people in disadvantaged rural communities that end up sourcing potentially sub-optimal drugs through these more loosely-regulated pathways (see Evans et al., 2019). Accelerating antimicrobial resistance (AMR) is another potential risk when the sales of antimicrobial drugs are inadequately controlled, however well-informed and well-intentioned individual retailers (like Isaac) may be.

## 6. Conclusion

Our work has revealed a deep-rooted tension: between a *principle-based*, *generalised ethical framework* that seeks to protect a public health good over the longer term, and a *pragmatic, contingent ethics-in-practice* that foregrounds immediate individual needs and accepts the reality of compromise. This tension arises directly from challenges in higher-level pharmaceutical governance that are, in turn, the product of under-resourced regulators and health systems operating within global supply chains that are difficult to regulate and where demand for affordable effective drugs far outstrips supply. In Ghana, the situation seems to be improving as regulatory capacity grows; in many other LMICs, the challenges remain as large as ever. As such, those working “on the ground” continue to bear the brunt of the ethical labour, working through complex bioethical conundrums case by case, relying on “hunches” and tacit understandings, carefully balancing legal, financial and moral risks.

Michael Lipksy’s seminal book on “street level bureaucrats” emphasises the central challenge to policy makers: “how to treat citizens alike in their claims on government and how at the same time to be responsive to the individual case when appropriate” (Lipsky, 2010: p.xii). The findings in this paper extend this challenge to how (and whether) the two ethical positions highlighted above can be reconciled so as not to deprive individuals urgently in need of essential medicines *now*, without perpetuating an iniquitous system that undermines, over the longer term, efforts to ensure high-quality healthcare for all.

An important first step is to recognise the constrained agency of those working on the ground who, because of challenges in higher-level pharmaceutical governance, have to make invidious decisions and compromises. They need access to information and advice that is *realistic* and recognises that compromises may sometimes need to be made. There have been some recent small-scale developments moves in this direction; for example, simplified checklists for frontline health-workers faced with medical products of uncertain quality (Schiavetti et al., 2020) and mobile phone app to facilitate reporting (Ciapponi et al., 2021).

Ultimately, though, the answer lies in addressing higher-level gaps in pharmaceutical governance: securing supply chains, improving distribution systems and regulating sales of controlled drugs. This will require increased global coordination, through efforts like the WHO’s Global Surveillance and Monitoring System (GSMS) for SF medical products, coupled with substantial scaling up of investment in health systems and NMRAs to narrow the gap between demand and supply of quality-assured essential medicines. This is no small task but the alternative is the continued delegation of ethical responsibility to those working on the “front line” and a tacit acceptance of “different [ethical] standards for the advantaged and the disadvantaged” (Farmer & Gastineau, 2004, p. 246).

## Notes

1 Our usage of the term “pharmaceutical governance” is thus broader than some other writers, who have focussed solely on corruption.

2 “Essential medicines” are defined as “those that satisfy the priority health care needs of a population” (WHO, n.d.). The WHO published its first Model List of Essential Medicines in 1977, with updates every two years.

3 In terminology agreed by the World Health Assembly, *substandard* medical products have been authorized by national authorities but fail to meet either quality standards or specifications, or both, whereas *“falsification”* refers to the deliberate (fraudulent) misrepresentation of a drug’s identity, composition, or source; a further concern is “unregistered/unlicensed” products, which have not been evaluated and/or approved by the relevant National or Regional Regulatory Authority.

4 Owing to participants’ concerns over the sensitivity of some of the material, we did not use audio recordings.

5 CHAG Official Website – Christian Health Association of Ghana

6 All names and certain details have been changed to protect participants’ anonymity.

7 At the time of fieldwork, there were more than 220 registered wholesalers operating in Ghana, compared with just 50 in the UK.

8 About 3 UK pence.
